# Perioperative lactate levels as prognostic indicators in patients undergoing early excision and grafting for extensive burns

**DOI:** 10.1186/s12893-025-03133-y

**Published:** 2025-08-31

**Authors:** Xinyi Li, Xiaodan Dong, Jianhua Luo, Lin Wang, Yang Cao, Yan Zhang, Bing Liang

**Affiliations:** Department of Anesthesiology, Guangzhou Red Cross Hospital of Jinan University, 396 Tongfu Middle Road, Guangzhou, 510220 China

**Keywords:** Extensive burns, Lactate, Prognosis, Early excision and grafting (EEG)

## Abstract

**Background:**

Elevated lactate levels are reliable biomarkers of tissue hypoperfusion and metabolic stress. However, their prognostic significance in extensive burn patients undergoing early excision and grafting (EEG) remains unclear. This study aimed to evaluate the prognostic value of perioperative lactate levels in predicting clinical deterioration following EEG in patients with extensive burns.

**Methods:**

In this retrospective cohort study, adult burn patients with ≥ 50% total burn surface area (TBSA) or ≥ 20% full-thickness burns treated between March 2021 and September 2023 were included. Patients were categorized into Deterioration and Non-Deterioration groups based on changes in Sequential Organ Failure Assessment (SOFA) scores. Statistical analyses included univariate and multivariate logistic regression, as well as receiver-operating characteristic (ROC) curve analysis.

**Results:**

Of 82 patients, 37.8% were classified into the Deterioration Group. Compared with the Non-Deterioration Group, these patients presented with a significantly higher burn index (BI), larger surgical area, and greater intraoperative blood transfusion volumes. Temporal trends in lactate levels appeared to differ between groups: lactate levels peaked on POD1 and returned to baseline by postoperative Day 3(POD3) in the Non-Deterioration Group, whereas they remained persistently elevated in the Deterioration Group. Among all perioperative time points, lactate levels on POD3 were significantly higher in the Deterioration Group (2.88 ± 0.65 mmol/L vs. 2.27 ± 0.68 mmol/L, *p* < 0.001) and were independently associated with clinical deterioration clinical deterioration (OR 2.97, 95% CI 1.18–8.71, *p* = 0.031). POD3 lactate levels demonstrated the highest discriminatory performance for identifying postoperative deterioration (AUC = 0.761, 95% CI 0.656–0.866).

**Conclusions:**

Among perioperative lactate levels in patients undergoing EEG for extensive burns, POD3 lactate levels were significantly associated with clinical deterioration and may serve as a useful marker to guide early risk assessment and supportive interventions.

## Introduction

Extensive burns, characterized by more than 50% total body surface area (TBSA) involvement or full-thickness burns covering at least 20% of the TBSA, are frequently accompanied by profound physiological disturbances. These include loss of skin barrier function, substantial fluid shifts, electrolyte imbalance, and heightened susceptibility to infection. In clinical observations, such injuries are often associated with systemic inflammatory responses and immune dysregulation, which may contribute to early multi-organ dysfunction in some patients [1]. The presence of necrotic tissue and eschar may further facilitate bacterial colonization and toxin release, potentially amplifying systemic inflammation and increasing the risk of clinical deterioration [2].

To mitigate these complications, early excision and grafting (EEG) has become a cornerstone of modern burn management [3]. Typically performed within several days following the resolution of the initial shock phase, EEG entails the removal of devitalized tissue and application of temporary wound coverage. This approach is intended to reduce microbial burden, attenuate systemic inflammation, and promote physiological stabilization [4]. Observational studies have reported associations between timely EEG and improved survival as well as shortened hospital stays [5]. However, in patients with extensive burns, the combination of ongoing fluid shifts, microcirculatory compromise, and systemic inflammatory responses may impair tissue perfusion and potentially reduce surgical tolerance [6]. Accurate perioperative risk stratification is therefore essential to guide individualized decision-making and optimize clinical outcomes [7].

Lactate is a well-established biomarker of tissue hypoperfusion and anaerobic metabolism, widely utilized in critical care to monitor the balance between oxygen delivery and consumption [8]. Elevated lactate levels have been consistently associated with increased mortality across various patient populations, including those with sepsis, trauma, and critical illness [9]. For example, a large cohort study reported that higher admission lactate levels in non-traumatic critically ill patients were significantly associated with increased 30-day mortality, reinforcing its potential value in outcome prediction [10]. In patients with sepsis, earlier lactate measurement has been linked to better risk stratification, whereas delayed assessment was associated with higher 28-day mortality [11]. In such clinical contexts, both absolute lactate values and temporal trends in lactate levels have demonstrated prognostic significance.

Despite frequent use in critical care, few studies have systematically examined how perioperative lactate levels correlate with early outcomes following major burn surgery. Existing research often lacks granularity in timing or fails to differentiate between transient postoperative elevation and sustained dysregulation [12]. Moreover, there is a paucity of data evaluating serial lactate trends in the context of EEG, a physiologically demanding intervention with high risk for postoperative complications [13]. This gap limits the application of lactate monitoring as a practical tool for early risk stratification in burn surgery. Therefore, we conducted a retrospective cohort study to assess the predictive value of perioperative lactate levels and provide insights to inform future interventions, potentially contribute to improved clinical decision-making.

## Materials and methods

### Study design and patient selection

This retrospective cohort study was conducted at Guangzhou Red Cross Hospital following approval from the Institutional Review Board of Guangzhou Red Cross Hospital (IRB No. 2021–2007-01). Data were retrieved from the medical records of adult burn patients treated between March 2021 and September 2023, with all information anonymized to maintain confidentiality in accordance with institutional and international ethical standards.

**Fig. 1:**
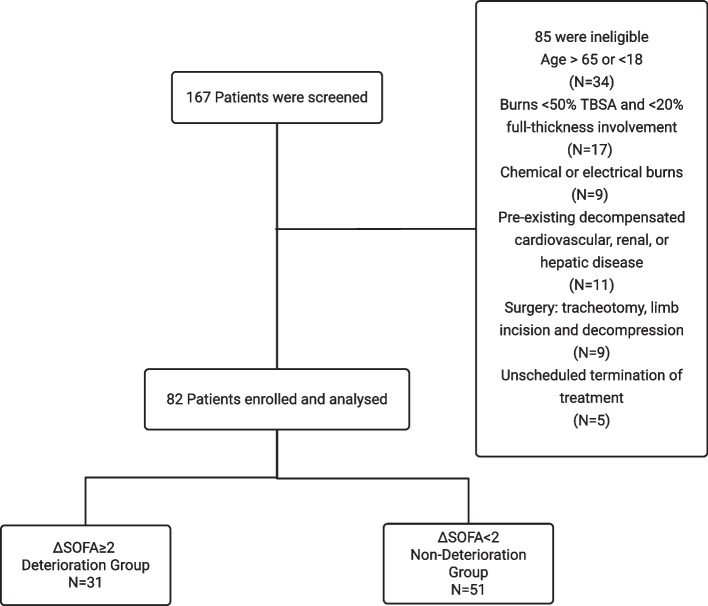
Flowchart of patient enrollment and exclusion criteria. Decompensated cardiovascular, renal, or hepatic diseases were defined based on NYHA class III–IV heart failure, chronic kidney disease stage ≥ 4, and Child–Pugh class B/C liver cirrhosis, respectively

Inclusion criteria were adult patients aged 18 to 65 with extensive thermal burns who underwent EEG, specifically defined as burns involving ≥ 50% TBSA or ≥ 20% full-thickness burns. Patients were excluded if they had severe pre-existing underlying conditions known to significantly impair perioperative tolerance or independently affect outcomes, operationally defined based on documented pre-admission medical history and physician assessment, including decompensated cardiovascular disease (New York Heart Association (NYHA) class III–IV heart failure) [14], advanced renal insufficiency (chronic kidney disease stage 4 or higher) [15], or hepatic dysfunction (Child–Pugh class B/C liver cirrhosis) [16]. Other exclusions included chemical or electrical burns, delayed or inadequate resuscitation, patients who underwent alternative surgical procedures, and those transferred or withdrawn prematurely from treatment, to minimize confounding (Fig. 1).

### Data collection and definition

Clinical data were retrospectively extracted from electronic medical records using a standardized abstraction protocol. All variables were cross-checked and underwent quality control procedures to enhance data accuracy and completeness prior to analysis.

#### Organ dysfunction assessment

The Sequential Organ Failure Assessment (SOFA) scores were obtained from the electronic medical records, where they had been originally assessed and documented by attending physicians as part of routine perioperative care. For this study, SOFA scores from the day before surgery (preoperative day) and postoperative day 1 (POD1) were retrieved. The SOFA system evaluates six organ systems: respiratory, coagulation, hepatic, cardiovascular, central nervous system, and renal [17]. Based on perioperative SOFA changes (ΔSOFA = POD1 SOFA – preoperative SOFA), patients were stratified into a Deterioration Group (ΔSOFA ≥ 2) and a Non-Deterioration Group (ΔSOFA < 2). This threshold was selected in reference to the Sepsis-3 criteria, which defines an acute increase in SOFA score by 2 points or more as indicative of clinically significant organ dysfunction [18].

#### Baseline and preoperative variables

Demographic and burn-specific variables recorded at admission comprised age, gender, body mass index (BMI), burn area (%TBSA), and burn index (BI). Burn severity was assessed on admission using the BI, calculated as %TBSA of full-thickness burns + 0.5 × %TBSA of partial-thickness burns [19]. Preoperative complications were defined as acute conditions that developed after burn injury and prior to the first EEG surgery. These included inhalation lung injury, acute respiratory distress syndrome (ARDS), renal insufficiency, and infection. Diagnoses were confirmed based on physician documentation in the electronic medical records, supported by relevant imaging studies, laboratory tests, and clinical progress notes. Infection was defined as a positive microbiological culture from wound exudate or blood, combined with at least one clinical or laboratory sign, including purulent wound discharge, leukocytosis, or new or worsening organ dysfunction [20]. The diagnoses of inhalation injury, ARDS, and renal insufficiency were based on established consensus definitions or widely accepted clinical criteria [21–23].

#### Physiological indicators and perioperative data

Preoperative physiological indicators included lactate levels, central venous oxygen saturation (ScvO_2_), and the venous-to-arterial carbon dioxide difference (Pcv-aCO_2_), along with the use of mechanical ventilation, and performance of tracheostomy. Intraoperative variables included the time from injury to EEG, surgical area, duration of surgery, operating room (OR) time, and graft type (xenograft or allograft). Intraoperative hemodynamic and fluid parameters included total input volume (fluid and transfusions), urine output, blood loss, and use of vasopressors. To account for body size, total fluid input was normalized to body weight (mL/kg), using the pre-injury or admission weight. Postoperative variables, such as lactate levels, ScvO_2_, and Pcv-aCO_2_, were measured on POD1 and POD3.

### Perioperative surgical protocol

#### Surgical timing and definition

Surgical excision remains a cornerstone of burn management. While EEG is generally recommended within 3–7 days post-injury to coincide with the resolution of burn shock [24–26], timing is often affected by hemodynamic instability, limited blood product availability, and socioeconomic factors. To reflect clinical practice variability, this study defined EEG as the first tangential excision procedure performed within 14 days of injury [27], provided the patient achieved stable vital signs.

#### Excision technique and wound coverage

During EEG, tangential excision was performed for full-thickness and deep partial-thickness burns, preserving viable dermis when feasible [25, 28]. Immediate autografting was not routine. Instead, wounds were covered using cryopreserved allografts or xenografts(primarily porcine) [29], selected based on patient financial capacity and institutional supply availability.

#### Hemostasis and temperature management

Hemostasis was achieved via tourniquets application(for limb), pre-incisional infiltration of diluted epinephrine solution (1:100,000), electrocautery, and postoperative pressure dressings [30–32]. To mitigate perioperative hypothermia, comprehensive temperature management was implemented: operating room temperature was maintained at approximately 32 °C, all intravenous and irrigation fluids were pre-warmed, and forced-air warming blankets were applied throughout surgery.

#### Intraoperative hemodynamic management

A goal-directed fluid strategy guided resuscitation, aiming to maintain adequate organ perfusion and urine output. Crystalloid solutions were administered based on intraoperative blood loss, hemodynamic changes, and urine output, with colloids added when indicated. Vasopressors were used selectively to maintain mean arterial pressure (MAP) above 65 mmHg when fluid therapy alone was insufficient.

#### Transfusion strategy

Red blood cell (RBC) transfusions were administered when intraoperative hemoglobin levels fell below 8 g/dL. Plasma transfusion was guided by cumulative blood loss, albumin concentrations, surgical extent, and hemodynamic instability [32–34]. Transfusion practices followed institutional protocols, accounting for both physiological thresholds and resource availability.

### Statistical methods

The sample size was determined using PASS 11 software. Based on a medium effect size (Cohen’s d = 0.5) derived from comparable studies, a minimum of 70 patients was required to achieve a power of 80% with a significance level of 0.05. To account for potential data loss, a 5% attrition rate was incorporated, resulting in a target sample size of 74 patients. Ultimately, 82 patients were enrolled to improve statistical robustness. The distribution of continuous variables was evaluated using the Shapiro–Wilk test to determine the suitability of parametric or non-parametric analysis methods. Categorical variables were compared between the Deterioration Group and Non-Deterioration Group using the Chi-square test or Fisher's exact test, while continuous variables were analyzed with Student's t-test or the Mann–Whitney U-test, as appropriate. Perioperative lactate levels (preoperative, POD1, and POD3) were compared within each group using the same approach based on data distribution. A multivariate logistic regression analysis with backwards stepwise regression was performed, including variables that demonstrated statistical significance in prior between-group comparisons. The Hosmer–Lemeshow test was used to assess model fitness, and Nagelkerke *R*^2^ was employed to evaluate model performance.

To assess the predictive values of preoperative and postoperative lactate levels as well as postoperative lactate clearance, receiver operating characteristic (ROC) curve analysis was performed. Optimal cut-off points were determined using the Youden index, and the sensitivity and specificity of these variables were calculated. The areas under the curve (AUCs) for preoperative and postoperative lactate levels were then compared to ascertain their relative predictive value. A *p*-value of < 0.05 was considered statistically significant. Statistical analyses were conducted using R software (version 4.3.2; R Foundation for Statistical Computing, Vienna, Austria). Data visualization and figure preparation were performed using GraphPad Prism (version 10.0, GraphPad Software, San Diego, CA, USA).

## Result

A total of 82 severely burned patients were included in this study. Based on changes in SOFA scores, 31 patients (37.8%) were classified into the Deterioration Group (ΔSOFA ≥ 2) and 51 patients (62.2%) into the Non-Deterioration Group (ΔSOFA<2). Compared to the Non-Deterioration Group, the Deterioration Group had a significantly higher BI (67.50 [42.50, 76.25] vs. 41.50 [31.00, 63.75], *p* = 0.027). In contrast, no significant differences were observed between the two groups regarding other baseline demographic and burn characteristics, or the incidence of preoperative complications (Table 1). In the group comparison of preoperative physiological indicators, Pcv-aCO2 levels were found to be significantly higher in the Deterioration Group compared to the Non-Deterioration Group (8.05 ± 1.48 mmHg vs. 7.34 ± 1.29 mmHg, *p* = 0.030) (Table 1).

**Table 1:** Baseline characteristics and preoperative physiological parameters

Characteristics	Deterioration group	Non-deterioration	*p*
	***N* = 31**	***N* = 51**	
Demographics			
Age (years)	44 (30.5, 50.0)	35 (31.0, 44.5)	0.384
Male, n (%)	27 (87.1%)	40 (78.43%)	0.490
BMI (kg/m^2^)	23.53 ± 2.31	23.11 ± 2.31	0.426
Burn Characteristics			
TBSA (%)	70 (52.5, 87.5)	60 (40.0, 82.5)	0.091
BI	67.50 (42.50, 76.25)	41.50 (31.00, 63.75)	0.027
Comorbidities, n (%)			
Inhalation lung injury	28 (90.32%)	50 (98.04%)	0.149
ARDS	4 (12.90%)	6 (11.76%)	1.000
Renal insufficiency	12 (38.71%)	18 (35.29%)	0.940
Infection	15 (48.39%)	17 (33.33%)	0.262
Physiological Indicators			
Lactate (mmol/L)	2.49 (2.00, 2.90)	2.22 (1.85, 2.53)	0.127
ScvO_2_(%)	75.85 ± 4.27	75.06 ± 3.98	0.409
Pcv-aCO_2_(mmHg)	8.05 ± 1.48	7.34 ± 1.29	0.030
Mechanical ventilation, n (%)	21 (67.74%)	28 (54.90%)	0.359
Tracheostomy, n (%)	21 (67.74%)	27 (52.94%)	0.277

Data are presented as mean ± SD, median (P25, P75), or number (%)

Definitions of comorbidities are provided in Section 2.2.2 of the Materials and Methods

*TBSA* Total body surface area, *BI* Burn index, *BMI* Body Mass Index, *ARDS* Acute Respiratory Distress Syndrome, *ScvO*_*2*_ Central venous oxygen saturation, *Pcv-aCO*_*2*_ Venous-to-arterial carbon dioxide difference

With regard to intraoperative factors, the Deterioration Group demonstrated a higher surgical area percentage (35% [27, 40] vs. 30% [20, 35], *p* = 0.031), greater intraoperative blood loss (400 [300, 600] mL vs. 300 [200, 400] mL, *p* = 0.046), and increased transfusion requirements, including RBC(4.00 [2.00, 4.75] units vs. 3.00 [2.00, 4.00] units, *p* = 0.014) and plasma (400 [200, 425] mL vs. 200 [0, 400] mL, *p* = 0.044). Although not statistically significant, total fluid input indexed to body weight was higher in the Deterioration Group (32.12 [23.56, 39.38] mL/kg vs. 26.68 [20.91, 33.07] mL/kg, p = 0.062). Regarding hemodynamic parameters, there were no significant differences in the vasopressor use (9.8% vs. 19.4%, *p* = 0.317) between the two groups (Table 2).

**Table 2:** Intraoperative factors

Characteristics	Deterioration group	Non-deterioration	*p*
	***N* = 31**	***N* = 51**	
Surgical Timing			
Time to EEG (days)	5 (4, 8)	6 (5, 8)	0.061
Surgical Parameters			
Surgical area (%)	35 (27, 40)	30 (20, 35)	0.031
Surgery duration (h)	3.08 (2.90, 3.72)	3.17 (2.71, 3.57)	0.599
OR time (h)	3.53 (3.13, 4.18)	3.58 (3.08, 4.08)	0.738
Graft type			
Xenograft, n (%)	8 (25.8%)	12 (23.5%)	1
Allograft, n (%)	23 (74.2%)	39 (76.5%)	1
Fluid Management			
RBC transfusion (units)	4.00 (2.00, 4.75)	3.00 (2.00, 4.00)	0.014
Plasma transfusion (ml)	400 (200, 425)	200 (0, 400)	0.044
Input per weight (ml/kg)	32.12 (23.56, 39.38)	26.68 (20.91, 33.07)	0.062
Urine output (ml)	300 (200, 400)	275 (200, 400)	0.691
Blood loss (ml)	400 (300, 600)	300 (200, 400)	0.046
Hemodynamic Monitoring			
Vasopressor use, n (%)	6(9.8%)	5(19.4%)	0.317

Data are presented as mean ± SD, median (P25, P75), or number (%)

*EEG* Early excision and grafting, *OR* Operating room, *RBC* Red blood cells

**Fig. 2:**
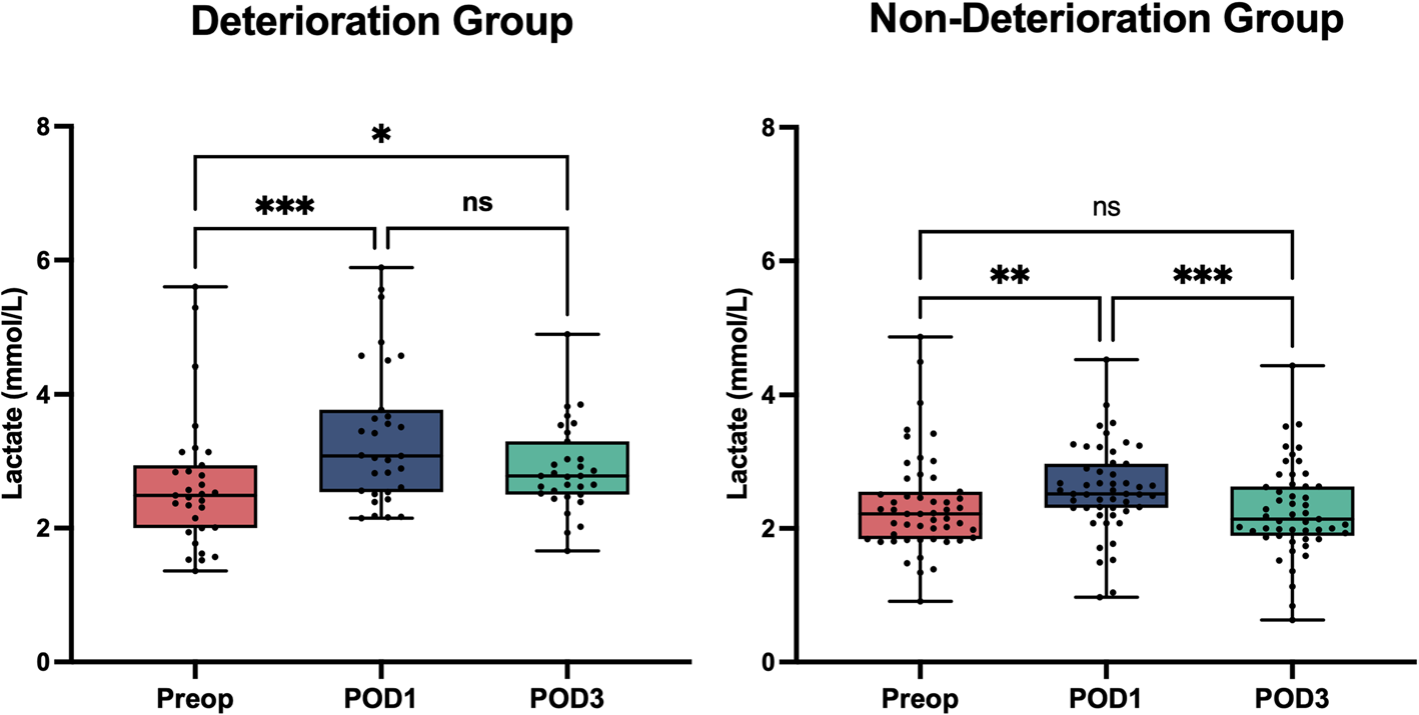
Temporal trends in perioperative lactate levels in Deterioration and Non-Deterioration groups. Lactate (mmol/L) were measured preoperatively (Preop), on postoperative Day 1 (POD1), and postoperative Day 3 (POD3). In the Deterioration group, lactate levels increased significantly on POD1 compared to preoperative baseline (*p* < 0.001), but no significant decline was observed by POD3 (ns vs. POD1), and levels remained significantly elevated relative to baseline (*p* < 0.05). In the Non-Deterioration group, lactate levels also increased on POD1 (*p* < 0.01 vs. Preop), but showed a significant decline by POD3 (*p* < 0.001 vs. POD1), returning to baseline levels (ns vs. Preop). Within-group comparisons were analyzed using the Wilcoxon signed-rank test. Significance levels are indicated as follows: *p < 0.05, **p < 0.01, **p < 0.001; ns = not significant

Postoperatively, POD1 lactate levels were significantly higher in the Deterioration Group compared to the Non-Deterioration Group (3.08 [2.55, 3.72] mmol/L vs. 2.52 [2.31, 2.94] mmol/L, *p* = 0.001), as were Pcv-aCO2 levels (7.80 ± 1.18 mmHg vs. 7.06 ± 1.03 mmHg, *p* = 0.005) (Table 5). POD3 lactate levels remained elevated in the Deterioration Group (2.88 ± 0.65 mmol/L vs. 2.27 ± 0.68 mmol/L, *p* < 0.001) (Table 3). Perioperative changes in lactate levels are illustrated in (Fig. 2). Within-group comparisons revealed that in the Deterioration group, lactate levels increase significantly on POD1 compared to preoperative levels (*p* < 0.001) but no significant decrease by POD3 (*p* > 0.05), with levels remaining significantly elevated relative to baseline (*p* < 0.05). In contrast, the Non-Deterioration group showed a comparable increased on POD1 (*p* < 0.01) but subsequently declined by POD3 (*p* < 0.01), returning to baseline levels (*p* > 0.05).

**Table 3:** Postoperative variables

Characteristics	Deterioration group	Non-deterioration	*p*
	***N* = 31**	***N* = 51**	
POD1			
Lactate (mmol/L)	3.08 (2.55, 3.72)	2.52 (2.31, 2.94)	0.001
ScvO_2_ (%)	73.50 (72.05, 77.05)	74.60 (72.55, 78.70)	0.284
Pcv-aCO_2_ (mmHg)	7.80 ± 1.18	7.06 ± 1.03	0.005
POD3			
Lactate (mmol/L)	2.88 ± 0.65	2.27 ± 0.68	< 0.001
ScvO_2_(%)	73.90 (72.10, 79.30)	75.80 (72.85, 78.30)	0.433
Pcv-aCO_2_(mmHg)	7.29 ± 1.01	6.99 ± 0.85	0.177

Data are presented as mean ± SD or median (P25, P75)

*ScvO*_*2*_ Central venous oxygen saturation, *Pcv-aCO*_*2*_ Venous-to-arterial carbon dioxide difference, *POD1* Postoperative Day 1; POD3: Postoperative Day 3

Multivariate analysis revealed that POD3 lactate level (OR 2.97, 95% CI 1.18–8.71, *p* = 0.031) had a significant effect on postoperative clinical outcomes. Furthermore, RBC transfusion (OR 1.40, 95% CI 0.98–2.04, *p* = 0.071), POD1 lactate level (OR 1.93, 95% CI 0.92–4.67, P = 0.109), and Pcv-aCO_2_ (OR 1.59, 95% CI 0.96–2.76, *p* = 0.082) demonstrated trends towards increased risk, though these trends did not reach statistical significance (Table 4). The model fitness was assessed by the Hosmer–Lemeshow test, which yielded an adequate result (*p* = 0.853). Nagelkerke *R*^2^ was 0.297.

**Table 4:** Multivariable logistic regression analysis of factors associated with postoperative clinical deterioration

Characteristics	B	SE	OR	OR (95% CI)	*p*
RBC transfusion (units)	0.33	0.18	1.40	(0.98, 2.04)	0.071
POD1					
Lactate (mmol/L)	0.66	0.41	1.93	(0.92, 4.67)	0.109
Pcv-aCO_2_ (mmHg)	0.46	0.27	1.59	(0.96, 2.76)	0.082
POD3					
Lactate (mmol/L)	1.09	0.51	2.97	(1.18, 8.71)	0.031

*B* Beta Coefficient, *SE* Standard Error, *OR* Odds Ratio, *CI* Confidence interval, *RBC* Red blood cells, *Pcv-aCO*_*2*_ Venous-to-arterial carbon dioxide difference, *POD1* Postoperative Day 1, *POD3* Postoperative Day 3

**Fig. 3:**
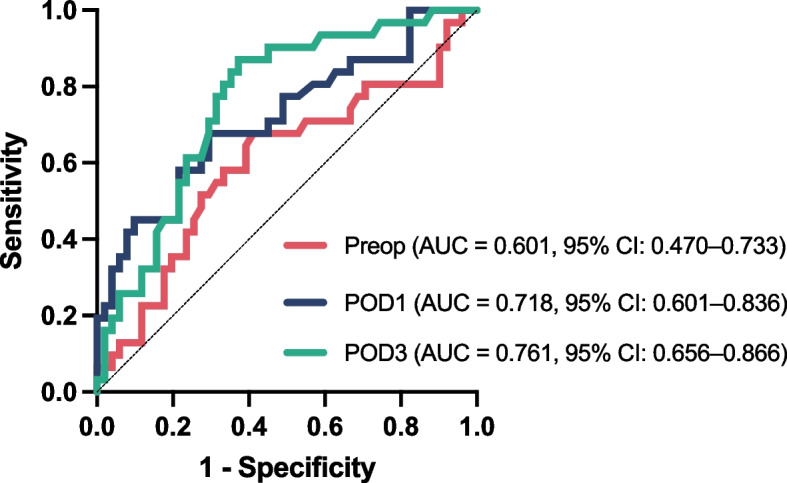
Receiver operating characteristic (ROC) curves for lactate (mmol/L) measured at three perioperative time points: preoperative (Preop, red), postoperative Day 1 (POD1, blue), and postoperative Day 3 (POD3, green). The area under the curve (AUC) was 0.601 for Preop lactate (95% confidence interval [CI]: 0.470–0.733), 0.718 (95% CI: 0.601–0.836) for POD1 lactate, and 0.761 (95% CI: 0.656–0.866) for POD3 lactate. The diagonal dashed line indicates the reference line for no discrimination (AUC = 0.5)

The AUC of the ROC curve analysis revealed that lactate levels on POD3 exhibited a higher discriminatory capacity (AUC: 0.761, 95% CI 0.656–0.866) in comparison to those on POD1 (AUC: 0.718, 95% CI 0.600–0.837) and preoperative levels (AUC: 0.601, 95% CI 0.469–0.734) (Fig. 3). The ROC curve suggests that lactate levels on POD3 exhibited the optimal discriminatory ability to predict the clinical outcome among the three time points. The optimal cut-off values for lactate levels were as follows: 2.300 for preoperative lactate, 2.815 for POD1 lactate, and 2.380 for POD3 lactate (Table 5).

**Table 5:** Receiver operating characteristic curve analysis

Characteristics	AUC	CI	Cut-off	Sensitivity	Specificity
Lactate (mmol/L)					
Preop	0.601	0.469–0.734	2.300	0.677	0.588
POD1	0.718	0.600–0.837	2.815	0.677	0.706
POD3	0.761	0.656–0.866	2.380	0.871	0.627

*AUC* Area under curve, *CI* Confidence interval, *Preop* Preoperative, *POD1* Postoperative Day 1, *POD3* Postoperative Day 3

## Discussion

This retrospective cohort study explored the association between perioperative lactate levels and postoperative clinical deterioration in patients undergoing EEG for extensive burns. Temporal patterns of lactate changes differed notably between groups: in the Non-Deterioration group, lactate levels peaked on POD1 but returned to baseline by POD3, whereas in the Deterioration group, levels did not exhibit a clear downward trend. These trends may indicate the potential utility of dynamic lactate monitoring for assessing recovery trajectories. Among all measured time points, lactate levels on POD3 showed the strongest association with adverse outcomes and identified as the only independent predictor in multivariate analysis. Collectively, these findings suggest that both the trajectory and timing of lactate changes may help inform early postoperative risk stratification in severely burned patients.

Lactate is a well-established biomarker of tissue hypoperfusion and anaerobic metabolism, reflecting the balance between oxygen delivery and consumption [8]. Previous studies have consistently reported associations between elevated preoperative lactate levels and adverse postoperative outcomes [35–37]. For instance, in patients undergoing extracorporeal membrane oxygenation (ECMO), higher preoperative lactate levels have been associated with prolonged recovery and increased length of hospital stay [38]. In our cohort, preoperative lactate levels were comparable between groups, which may reflect the effects of early standardized fluid resuscitation in stabilizing hemodynamics prior to surgery [39]. While such stabilization may indicate a transition beyond the acute stress phase, subsequent lactate dynamics could reflect the disturbances introduced by surgical intervention.

In the postoperative period, lactate dynamics are shaped by multiple interacting factors, including surgical trauma, systemic inflammation, and temporary alterations in tissue perfusion [40]. During EEG, eschar removal reduces necrotic tissue burden and bacterial load but also disrupts the temporary metabolic equilibrium, releasing inflammatory mediators and toxins that may exacerbate systemic inflammatory response syndrome (SIRS) and tissue hypoxia [13, 41]. These acute physiologic disturbances could contribute to transient hyperlactatemia shortly after surgery. In our cohort, lactate levels were elevated on POD1 in both groups, but their limited discriminatory value suggests that such early measurements may predominantly reflect short-term metabolic stress rather than sustained dysfunction. Patients in the Deterioration group experienced a more substantial intraoperative burden, reflected by greater surgical area, increased blood loss, and higher transfusion requirements, and a trend toward increased fluid input per kilogram. These factors, including both surgical trauma and hemodynamic management, might have contributed to transient lactate elevations by increasing metabolic stress and impairing oxygen delivery [35]. These transient perioperative disturbances could compromise the interpretability of early lactate measurements by masking the patient’s true physiological condition. Thus, POD1 lactate levels may reflect transient surgical stress responses more than sustained physiological derangement, limiting their value as a standalone prognostic indicator.

In contrast, lactate levels measured on POD3 appear less influenced by transient perioperative disturbances and may more accurately reflect the patient’s systemic recovery trajectory. At this stage, elevated lactate concentrations may indicate unresolved hypoperfusion, sustained inflammation, or the early organ dysfunction, particularly involving hepatic or renal pathways [42]. In our analysis, POD3 lactate levels not only differed significantly between groups but also emerged as the only independent predictor of clinical deterioration in multivariable modeling. This supports the interpretation that POD3 values may better capture ongoing physiological imbalance beyond the acute perioperative phase. Moreover, the within-group persistence of elevated lactate levels in the Deterioration group, as demonstrated by within-group comparisons (Fig. 2), reinforces its potential role as a stable biomarker. These findings are consistent with previous findings in critical care settings, where delayed lactate clearance has been associated with worse outcomes [43, 44]. Taken together, these results suggest that while early postoperative values may reflect acute surgical impact, POD3 lactate levels may offer greater prognostic value for identifying patients at risk of clinical decline following major burn surgery.

In addition to intraoperative factors, the underlying severity of burn injury likely contributes to postoperative lactate dynamics. Notably, although the TBSA did not differ significantly between groups, the Deterioration group exhibited a markedly higher BI, indicating more extensive full-thickness involvement [45]. BI integrates both surface area and depth of injury, providing a more comprehensive indicator of burn severity than TBSA alone [6]. Deeper burns are associated with heightened systemic inflammation, increased capillary permeability, and impaired tissue perfusion, all of which may contribute to delayed lactate clearance [46]. These physiological burdens may explain the persistent elevation of POD3 lactate levels observed in the Deterioration Group despite similar preoperative values. Such observations suggest that burn depth, as quantified by BI, may influence the duration of postoperative metabolic imbalance and the trajectory of lactate normalization. This underscores the potential utility of BI in interpreting lactate trends and informing early postoperative risk stratification in the early postoperative period.

While variables such as POD1 lactate levels, Pcv-aCO_2_ and RBC transfusion demonstrated trends toward worse outcomes in the multivariable model, none reached statistical significance. This lack of significance may reflect the limitations of sample size or residual confounding from variability in perioperative management. Notably, POD1 lactate levels were elevated in both groups but exhibited limited discriminatory power. This may be due to their susceptibility to transient intraoperative perturbations, such as fluid shifts, blood loss, and hemodynamic instability, which can elevate lactate without necessarily indicating persistent metabolic stress [35]. These observations underscore the need for cautious interpretation of early postoperative lactate values, particularly when used in isolation. Although intraoperative indicators such as vasopressor use did not significantly differ between groups, a non-significant trend toward greater fluid input per kilogram was observed in the Deterioration Group. While not statistically conclusive, these variables remain physiologically relevant and may influence lactate kinetics in the perioperative setting [47]. Their incorporation into future prospective studies, particularly those employing continuous hemodynamic monitoring and serial lactate profiling, may help elucidate their independent prognostic value.

Although these findings contribute to our understanding of perioperative lactate dynamics, their interpretation must be viewed in light of several limitations. First, its retrospective single-center design and modest sample size may limit causal inference and reduce generalizability. Second, while intraoperative variables such as vasopressor use were reported, continuous hemodynamic monitoring and vasopressor dosage data were unavailable, restricting more granular analysis of perfusion dynamics. Third, although standardized intraoperative temperature management protocols were implemented, continuous core temperature data was not performed, which may have constrained on lactate levels. Fourth, long-term outcomes such as mortality and ICU stay were not included, as this study focused on short-term organ dysfunction. Finally, perioperative management practices was not fully standardized across all cases, which may introduce unmeasured confounding despite adherence to institutional protocols.

## Conclusion

In conclusion, this retrospective cohort study found that lactate levels on POD3 were significantly associated with subsequent clinical deterioration in patients undergoing EEG for extensive burns. Compared to earlier measurements, POD3 lactate values may be less influenced by transient intraoperative disturbances and could more accurately reflect ongoing physiological recovery. Serial lactate monitoring, particularly with attention to temporal trends, may support early postoperative risk assessment and clinical decision-making. However, given the observational design and sample size limitations, these findings should be interpreted with caution, and further prospective studies are needed to confirm the prognostic value of POD3 lactate and evaluate the broader role of dynamic lactate profiling in burn care.

## Data Availability

Data of the current study are available from the corresponding author on reasonable request.

## Abbreviations

AUC: Areas under the curve
BI: Burn index
BMI: Body mass index
ECMO: Extracorporeal membrane oxygenation
EEG: Early excision and grafting
MAP: Mean arterial pressure
MODS: Multi-organ dysfunction syndrome
OR: Operating room
Pcv-aCO_2_: Venous-to-arterial carbon dioxide difference
Preop: Preoperative
POD1: Postoperative day 1
POD3: Postoperative day 3
RBC: Red blood cells
ROC: Receiver-operating characteristic
ScvO_2_: Central venous oxygen saturation
SIRS: Systemic inflammatory response syndrome
SOFA: Sequential Organ Failure Assessment
TBSA: Total body surface area

## References

[1] Vinaik R, Barayan D, Jeschke MG. Pathophysiology and hypermetabolic response to burn. In: Lee JO, editor. Essential burn care for non-burn specialists. Cham: Springer International Publishing; 2023. p. 29–84. 10.1007/978-3-031-28898-2_2.

[2] D’Abbondanza JA, Shahrokhi S. Burn infection and burn sepsis. Surg Infect. 2021.10.1089/sur.2020.102. 10.1089/sur.2020.102 32364824

[3] Hoogewerf CJ, et al. Early excision and grafting for burns. Cochrane Database Syst Rev. 2012;2012:CD009715.

[4] Greenhalgh DG. Management of burns. N Engl J Med. 2019;380:2349–59. 31189038 10.1056/NEJMra1807442

[5] Glaser J, et al. The status quo of early burn wound excision: insights from the German burn registry. Burns. 2021;47:1259–64. 34330580 10.1016/j.burns.2021.06.010

[6] Jeschke MG, et al. Burn injury. Nat Rev Dis Primer. 2020;6:11. 10.1038/s41572-020-0145-5 PMC7224101 32054846

[7] Ong YS, Samuel M, Song C. Meta-analysis of early excision of burns. Burns. 2006;32:145–50. 16414197 10.1016/j.burns.2005.09.005

[8] Brooks GA. Lactate as a fulcrum of metabolism. Redox Biol. 2020;35:101454. 32113910 10.1016/j.redox.2020.101454 PMC7284908

[9] Liu Z, et al. Prognostic accuracy of the serum lactate level, the SOFA score and the qSOFA score for mortality among adults with sepsis. Scand J Trauma Resusc Emerg Med. 2019;27:51. 31039813 10.1186/s13049-019-0609-3 PMC6492372

[10] Bernhard M, et al. Elevated admission lactate levels in the emergency department are associated with increased 30-day mortality in non-trauma critically ill patients. Scand J Trauma Resusc Emerg Med. 2020;28:82. 32807232 10.1186/s13049-020-00777-y PMC7433202

[11] Chen H, Zhao C, Wei Y, Jin J. Early lactate measurement is associated with better outcomes in septic patients with an elevated serum lactate level. Crit Care. 2019;23:351. 31711512 10.1186/s13054-019-2625-0 PMC6849274

[12] Cartotto R, et al. American burn association clinical practice guidelines on burn shock resuscitation. J Burn Care Res. 2024;45:565–89. 38051821 10.1093/jbcr/irad125

[13] Kallinen O, Maisniemi K, Böhling T, Tukiainen E, Koljonen V. Multiple organ failure as a cause of death in patients with severe burns. J Burn Care Res. 2012;33:206–11. 21979843 10.1097/BCR.0b013e3182331e73

[14] Caraballo C, et al. Clinical implications of the New York Heart Association classification. J Am Heart Assoc. 2019;8:e014240. 31771438 10.1161/JAHA.119.014240 PMC6912957

[15] Levin A, Stevens PE. Summary of KDIGO 2012 CKD guideline: behind the scenes, need for guidance, and a framework for moving forward. Kidney Int. 2014;85:49–61. 24284513 10.1038/ki.2013.444

[16] Tsoris A, Marlar CA. Use of the child pugh score in liver disease. Treasure Island (FL): In StatPearls. StatPearls Publishing; 2025. 31194448

[17] Vincent JL, et al. The SOFA (sepsis-related organ failure assessment) score to describe organ dysfunction/failure. On behalf of the working group on sepsis-related problems of the european society of intensive care medicine. Intensive Care Med. 1996;22:707–10. 8844239 10.1007/BF01709751

[18] Singer M, et al. The third international consensus definitions for sepsis and septic shock (sepsis-3). JAMA. 2016;315:801–10. 26903338 10.1001/jama.2016.0287 PMC4968574

[19] Sheppard NN, Hemington-Gorse S, Shelley OP, Philp B, Dziewulski P. Prognostic scoring systems in burns: a review. Burns. 2011;37:1288–95. 21940104 10.1016/j.burns.2011.07.017

[20] Greenhalgh DG. Sepsis in the burn patient: a different problem than sepsis in the general population. Burns Trauma. 2017;5:23. 28795054 10.1186/s41038-017-0089-5 PMC5547526

[21] Walker PF, et al. Diagnosis and management of inhalation injury: an updated review. Crit Care. 2015;19:351. 26507130 10.1186/s13054-015-1077-4 PMC4624587

[22] ARDS Definition Task Force, et al. Acute respiratory distress syndrome: the berlin definition. JAMA. 2012;307:2526–33. 22797452 10.1001/jama.2012.5669

[23] James M, et al. Canadian society of nephrology commentary on the 2012 KDIGO clinical practice guideline for acute kidney injury. Am J Kidney Dis. 2013;61:673–85. 23518195 10.1053/j.ajkd.2013.02.350

[24] Lee JO, et al. Operative wound management. Total Burn Care (Elsevier Inc.). 2012:157-172.e2.10.1016/B978-1-4377-2786-9.00013-8.

[25] Song G, et al. Experience and efficacy of surgery for retaining viable subcutaneous tissue in extensive full-thickness burns. Burns. 2016;42:71–80. 26546384 10.1016/j.burns.2015.06.012

[26] Song G, Shi W. Early excision and skin grafting for extensive deep burns. Chin J Injury Repair (Electronic Edition). 2017;12:56.

[27] Ayaz M, Keshavarzi A, Bahadora H, Arasteh P, Moslemi S. Comparison of the results of early excision and grafting between children and adults; a prospective comparative study. Bull Emerg Trauma. 2017;5:179–83. 28795062 PMC5547205

[28] Anyanwu JA, Cindass R. Burn debridement, grafting, and reconstruction. In: StatPearls [Internet]. Treasure Island (FL): StatPearls Publishing; 2023–. Available from: https://www.ncbi.nlm.nih.gov/books/NBK551717/. 31869181

[29] Radzikowska-Büchner E, et al. An overview of recent developments in the management of burn injuries. Int J Mol Sci. 2023;24:16357. 38003548 10.3390/ijms242216357 PMC10671630

[30] Chih-chun Y, Tsi-siang S, Wei-shia X. A Chinese concept of treatment of extensive third-degree burns. Plast Reconstr Surg. 1982;70:238–54. 7048371 10.1097/00006534-198208000-00025

[31] Desai MH, et al. Early burn wound excision significantly reduces blood loss. Ann Surg. 1990;211:753–62. 2357138 10.1097/00000658-199006000-00015 PMC1358131

[32] Cartotto R, Musgrave MA, Beveridge M, Fish J, Gomez M. Minimizing blood loss in burn surgery. J Trauma. 2000;49:1034–9. 11130485 10.1097/00005373-200012000-00010

[33] Li P, et al. Blood loss and influencing factors during tangential excision and grafting in patients with extensive deep burns. Chin J Burns Wound Repair. 2017;33:111–4.

[34] Orgill DP. Excision and skin grafting of thermal burns. N Engl J Med. 2009;360:893–901. 19246361 10.1056/NEJMct0804451

[35] Sánchez M, et al. A protocol for resuscitation of severe burn patients guided by transpulmonary thermodilution and lactate levels: a 3-year prospective cohort study. Crit Care. 2013;17:R176. 23947945 10.1186/cc12855 PMC4057032

[36] Muthukumar V, et al. Blood lactate and lactate clearance: Refined biomarker and prognostic marker in burn resuscitation. Ann Burns Fire Disasters. 2020;33:293–8. 33708018 PMC7894842

[37] Jones AE. Lactate clearance vs central venous oxygen saturation as goals of early sepsis therapyA randomized clinical trial. JAMA. 2010;303:739. 20179283 10.1001/jama.2010.158 PMC2918907

[38] Mungan İ, Kazancı D, Bektaş Ş, Ademoglu D, Turan S. Does lactate clearance prognosticates outcomes in ECMO therapy: a retrospective observational study. BMC Anesthesiol. 2018;18(1):152. 10.1186/s12871-018-0618-1. 10.1186/s12871-018-0618-1 PMC6201528 30355289

[39] Bateman RM, et al. 36th international symposium on intensive care and emergency medicine. Crit Care. 2016;20:94. 27885969 10.1186/s13054-016-1208-6 PMC5493079

[40] Carmona P, et al. Hyperlactatemia in surgical ablation of atrial fibrillation and cardiac surgery. Is it a predictive factor of postoperative morbidity? Rev Esp Anestesiol Reanim. 2014;61:311–8. 24556510 10.1016/j.redar.2014.01.003

[41] Khuchua E, Didbaridze T, Ormotsadze G, Sanikidze T, Pachkoria E, Ratiani L, Gvajaia N, Kupradze V. Evaluating the diagnostic and prognostic value of interleukin-6 (IL-6) and soluble triggering receptor expressed on myeloid cells-1 (sTREM-1) in systemic inflammatory response syndrome (SIRS) and sepsis in adults. Cureus. 2024;16(11):e73310. 10.7759/cureus.73310. 10.7759/cureus.73310 PMC11626217 39655134

[42] Kiyatkin ME, Bakker J. Lactate and microcirculation as suitable targets for hemodynamic optimization in resuscitation of circulatory shock. Curr Opin Crit Care. 2017;23(4):348–54. 28537998 10.1097/MCC.0000000000000423

[43] Nguyen HB, et al. Early lactate clearance is associated with improved outcome in severe sepsis and septic shock. Crit Care Med. 2004;32:1637. 15286537 10.1097/01.ccm.0000132904.35713.a7

[44] Jansen TC, et al. Early lactate-guided therapy in intensive care unit patients. Am J Respir Crit Care Med. 2010;182:752–61. 20463176 10.1164/rccm.200912-1918OC

[45] Anonymous. Dynamic monitoring and analysis of blood lactate levels in burn patients. Chin J Burns. 2008;24:61.

[46] Nielson CB, Duethman NC, Howard JM, Moncure M, Wood JG. Burns: pathophysiology of systemic complications and current management. J Burn Care Res. 2017;38:e469–81. 27183443 10.1097/BCR.0000000000000355 PMC5214064

[47] Myburgh JA, Mythen MG. Resuscitation fluids. N Engl J Med. 2013;369:1243–51. 24066745 10.1056/NEJMra1208627

